# Maize FERONIA‐like receptor genes are involved in the response of multiple disease resistance in maize

**DOI:** 10.1111/mpp.13232

**Published:** 2022-05-21

**Authors:** Haiyue Yu, Hongchun Ruan, Xinyao Xia, Aline Sartor Chicowski, Steven A. Whitham, Zhiqiang Li, Guirong Wang, Wende Liu

**Affiliations:** ^1^ Shenzhen Branch, Guangdong Laboratory of Lingnan Modern Agriculture, Genome Analysis Laboratory of the Ministry of Agriculture Agriculture Genomics Institute at Shenzhen, Chinese Academy of Agricultural Sciences Shenzhen China; ^2^ State Key Laboratory for Biology of Plant Diseases and Insect Pests Institute of Plant Protection, Chinese Academy of Agricultural Sciences Beijing China; ^3^ Institute of Plant Protection Fujian Academy of Agricultural Sciences Fuzhou China; ^4^ Department of Plant Pathology and Microbiology Iowa State University Ames Iowa USA

**Keywords:** cell death, FERONIA, FLR, maize disease, reactive oxygen species (ROS), virus‐induced gene silencing (VIGS)

## Abstract

Receptor‐like kinases (RLKs) are key modulators of diverse cellular processes such as development and sensing the extracellular environment. FERONIA, a member of the *Cr*RLK1L subfamily, acts as a pleiotropic regulator of plant immune responses, but little is known about how maize *FERONIA‐like* receptors (*FLR*s) function in responding to the major foliar diseases of maize such as northern corn leaf blight (NLB), northern corn leaf spot (NLS), anthracnose stalk rot (ASR), and southern corn leaf blight (SLB). Here, we identified three ZmFLR homologous proteins that showed cell membrane localization. Transient expression in *Nicotiana benthamiana* proved that ZmFLRs were capable of inducing cell death. To investigate the role of *ZmFLR*s in maize, we used virus‐induced gene silencing to knock down expression of *ZmFLR1/2* and *ZmFLR3* resulting in reduced reactive oxygen species production induced by flg22 and chitin. The resistance of maize to NLB, NLS, ASR, and SLB was also reduced in the *ZmFLR*s knockdown maize plants. These results indicate that *ZmFLR*s are positively involved in broad‐spectrum disease resistance in maize.

## Graphical abstract

Maize FERONIA‐like receptor genes are involved in broad‐spectrum disease resistance in maize, positively regulating resistance to northern corn leaf blight and leaf spot, anthracnose stalk rot, and southern corn leaf blight.

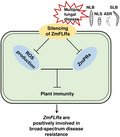

## 1 INTRODUCTION

Maize (*Zea mays*) is one of the most important crops worldwide, being used as human food, livestock feed, and export. Different maize diseases can cause yield and quality losses, which is a major threat to the economy and food security worldwide (Balint‐Kurti & Johal, 2009). Northern corn leaf blight (NLB), southern corn leaf blight (SLB), northern corn leaf spot (NLS), and anthracnose stalk rot (ASR) are major maize foliar diseases throughout the world (Dai et al., 2018; Liu et al., 2015; Mueller et al., 2016). NLB is caused by the hemibiotrophic fungus *Setosphaeria turcica*, NLS is caused by *Bipolaris zeicola*, ASR is caused by *Colletotrichum graminicola*, and SLB is caused by the necrotrophic fungus *Bipolaris maydis*. Both NLB and ASR rank among the most devastating maize fungal diseases in the United States and Canada, causing yield losses of more than 40% in conducive environmental conditions (Mueller et al., 2016). Furthermore, due to changes in cultivation strategies, climate, and the extensive use of susceptible maize hybrids, NLB and ASR have the potential to cause serious yield losses in maize production in countries such as China and Brazil, the second and third largest producers of maize in the world, respectively (FAO, 2019). In addition, *B. zeicola* and *B. maydis* are the major pathogens affecting maize production in China (Dai et al., 2018; Liu et al., 2015). NLS and SLB can cause yield losses of 10%–20% in years with severe epidemics (Dai et al., 2016; Sun et al., 2020).

Higher plants possess a two‐layer immune system to sense varieties of immunogenic signals when infected with fungal pathogen (Boller & He, 2009). Cell‐surface pattern recognition receptors (PRRs) typically perceive pathogen‐/damage‐associated molecules or apoplastic pathogen‐associated effectors (Boutrot & Zipfel, 2017; Couto & Zipfel, 2016; Yu et al., 2017). Intracellular receptors, most commonly nucleotide‐biding leucine‐rich repeat proteins (NLRs), sense pathogen effectors that are delivered into the plant cell (Wu et al., 2017). Prior research generally confirms that a variety of RLKs, such as leucine‐rich repeat RLKs, cell wall‐associated RLKs, lectin RLKs, proline‐rich extension‐like RLKs, and *Catharanthus roseus* RLK1‐like kinases (*Cr*RLK1Ls), regulate many cellular processes during vegetative and reproductive development (Dievart & Clark, 2004; Escobar‐Restrepo et al., 2007; Jose et al., 2020; Ringli, 2010). *Cr*RLK1 was first isolated from a suspension of cells of *Catharanthus roseus*, and is a receptor‐like protein kinase (Schulze‐Muth et al., 1996). *Cr*RLK1Ls are involved in many processes, such as cellular growth and morphogenesis, reproduction, immunity, hormone signalling, and abiotic stress tolerance (Franck et al., 2018). In *Arabidopsis*, all 17 members of the *Cr*RLK1L subfamily possess an extracellular domain (ECD) with two malectin‐like domains (MLD), a transmembrane domain, and an intracellular serine/threonine kinase domain (Lindner et al., 2012). The gene encoding FERONIA (FER), a well‐characterized member of the *Cr*RLK1Ls subfamily, was first cloned during the screening of double‐fertilization regulators participating in pollen tube reception through reactive oxygen species (ROS) and Ca^2+^ signalling (Escobar‐Restrepo et al., 2007). FER is also involved in cell growth. A FER loss‐of‐function mutant showed obvious root hair defects (Duan et al., 2010), severe hypocotyl inhibition (Deslauriers & Larsen, 2010), and severe cell elongation defects (Guo et al., 2009). In rice, two homologous *FERONIA*‐*like receptors* (*FLRs*) were shown to control plant morphology, fertility, and seed yield (Li et al., 2016). Furthermore, FER participates in a variety of plant hormone responses. FER employs the small G protein signalling network mediated by GEF1/4/10‐ROP11 to directly activate the phosphatase activity of the key regulator ABI2 in the abscisic acid (ABA) signalling pathway, thereby negatively regulating the ABA response (Yu et al., 2012). In contrast, auxin is positively regulated by FER through the GRE‐ROP/ARAC module (Duan et al., 2010). Moreover, the FER‐dependent brassinosteroid (BR) response exhibits an antagonistic effect with ethylene on hypocotyl shortening (Deslauriers & Larsen, 2010). Additionally, FER negatively regulates S‐adenosylmethionine (SAM) synthesis by interacting with SAM synthases (SAM1 and SAM2), thereby inhibiting ethylene production (Mao et al., 2015). FER has also been shown to positively regulate immunity by inhibiting jasmonic (JA) acid and coronatine (COR) signalling in *Arabidopsis* (Guo et al., 2018).

FER also works as a prominent component in the plant immune response. *Arabidopsis* plants display enhanced resistance to the fungal pathogens *Fusarium oxysporum* and *Golovinomyces* (syn. *Erysiphe*) *orontii* in the absence of FER (Kessler et al., 2010; Masachis et al., 2016). In parallel, *FLR2* and *FLR11* mutations lead to increased resistance to *Magnaporthe oryzae* without growth penalty in rice plants (Yang et al., 2020). However, the *Arabidopsis fer* mutant was more susceptible to *Hyaloperonospora arabidopsidis* and *Colletotrichum higgansianum* (Kessler et al., 2010). Prior research has thoroughly investigated the role of FER in modulating the receptor kinase complex assembly, and its influence on pathogen‐associated molecular pattern (PAMP)‐triggered immunity (PTI), being required for the ROS burst triggered by flg22 and chitin (Stegmann et al., 2017). FER promotes the association of FLS2‐BAK1 complexes and EFR‐ BAK1 complexes in response to flg22 and elf18, respectively (Stegmann et al., 2017). ANXUR1 (ANX1) and ANXUR2 (ANX2), which have extremely high sequence similarity to FER, can also directly bind with the FLS2‐BAK1 complex but negatively regulate PTI (Mang et al., 2017). Soybean (*Glycine max*) also harbours a similar module, the malectin‐like receptor kinase GmLMM1, that serves as a molecular adjustor in regulating immune activation (Wang et al., 2020). These findings illustrated that FER manages diverse cellular processes in response to different pathogens, but little is known about how FER works against maize fungal diseases. To further understand the effect of *FERONIA‐like receptor* genes in the response to multiple diseases in maize, we characterized three *AtFER* homologues: ZmFLR1 (Zm00001d047533), ZmFLR2 (Zm00001d029047), and ZmFLR3 (Zm00001d002175). All three maize proteins were membrane localized and were able to cause plant cell death. Furthermore, we generated virus‐induced gene silenced (VIGS) maize plants FoMV:FLR1/2 and FoMV:FLR3, which had altered response to chitin and flg22, and enhanced susceptibility to *S. turcica*, *B. zeicola*, *C. graminicola*, and *B. maydis*. Our data will contribute to explaining the function of *ZmFLR* genes regulating maize resistance to *S. turcica*, *B. zeicola*, *C. graminicola*, and *B. maydis*, providing a theoretical basis for designing targeted intervention strategies to generate disease‐resistant maize plants.

## 2 RESULTS

### 2.1 Identification of FLR homologues in maize

**FIGURE 1:**
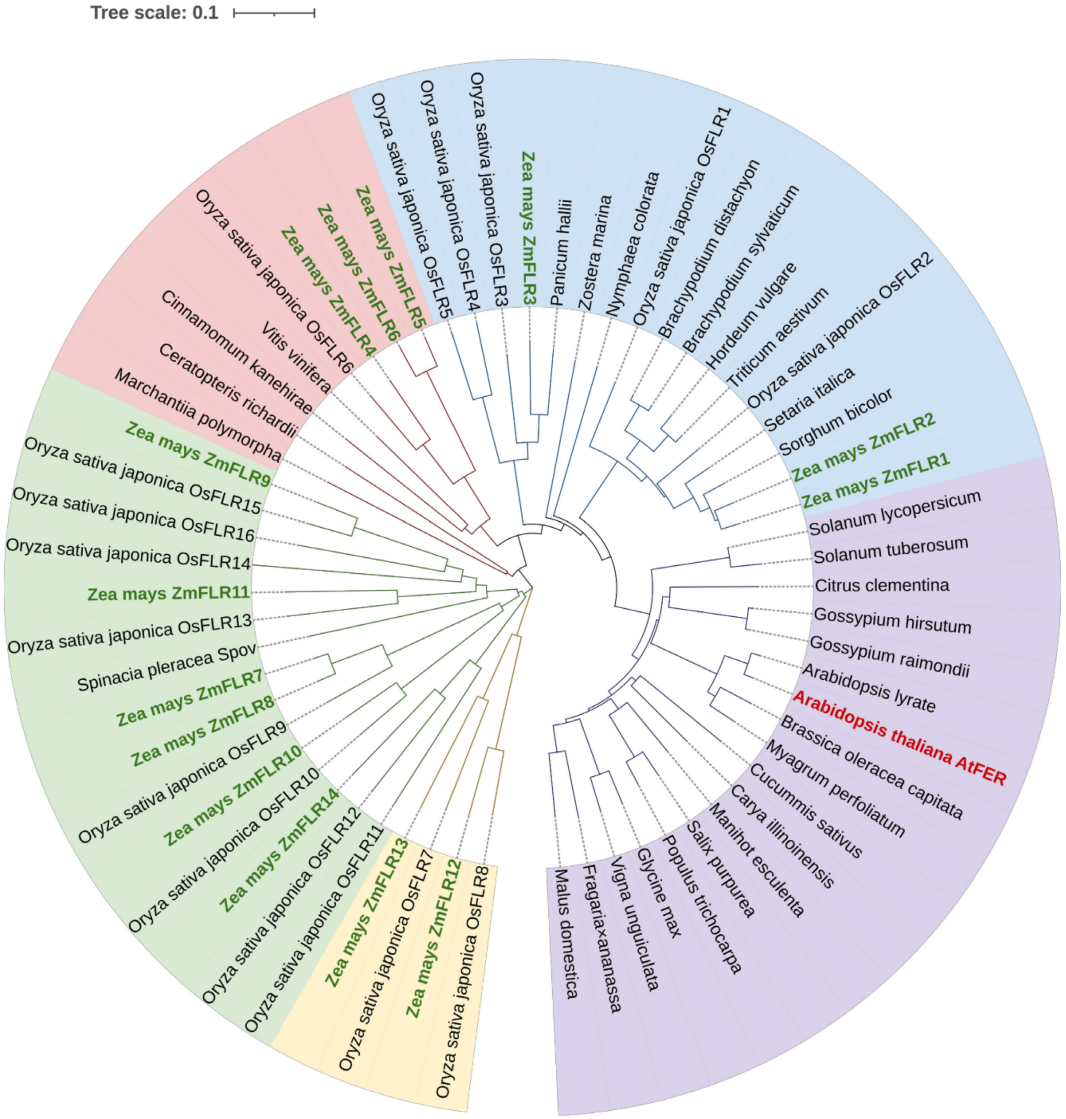
Phylogenetic tree of the *Cr*RLK1L family proteins. Full‐length amino acid sequences of 62 FER homologues from 35 diverse plant species were used to construct the phylogenetic tree via the neighbour‐joining method with 1000 bootstrap values in MEGA 7 and was optimized with the iTOL online tool. The analysed *Cr*RLK1Ls were classified into five subgroups, I–V, marked with different background colours. AtFER is highlighted in red and ZmFLR1, ZmFLR2, and ZmFLR3 are highlighted in green (Zm, *Zea mays*).

*Arabidopsis* FERONIA (*AtFER*) is a representative member of the *CrRLK1L* subfamily and is evolutionarily conserved. The amino acid sequence of AtFER was used for BLAST analysis in Phytozome (https://phytozome.jgi.doe.gov/pz/portal.html) to determine whether AtFER homologous proteins exist in maize. Our search identified 14 *Cr*RLK1L subfamily members in maize. We compared the amino acid sequences of 62 FER homologues in various plant species, which included AtFER, 14 maize putative *Cr*RLK1Ls, 16 rice OsFLRs, and other 31 FER homologues from various plant species to explore the evolutionary relationship of FERs. The neighbour‐joining method was used to construct a phylogenetic tree (Figure 1). Two lower plant species, *Marchantia polymorpha* and *Ceratopteris richardii*, were chosen to represent mosses and pteridophytes, respectively. According to the phylogenetic tree, the 62 FER homologues could be classified into five subgroups (subgroups I–V) comprising 18 members in group I, 17 members in group II, 8 members in group III, 15 members in group IV, and 4 members in group V (Figure 1). Group I included AtFER, and it was mainly composed of dicotyledonous plants, which suggests that the FER of dicotyledonous plants is evolutionary conserved. The phylogenetic analysis of the evolutionary relationship of FERs in different species indicated that group II included three highly similar maize RLK gene homologues encoding FERONIA‐like receptor (FLRs), named ZmFLR1 (Zm00001d047533, sharing 73.6% amino acid identity with AtFER), ZmFLR2 (Zm00001d029047, sharing 67.5% amino acid identity with AtFER), and ZmFLR3 (Zm00001d002175, sharing 66.4% amino acid identity with AtFER). Among the 14 ZmFLRs, ZmFLR1, ZmFLR2, and ZmFLR3 have the closest evolutionary distances to AtFER, 0.339, 0.341 and 0.439, respectively (Table S2). These three highly homologous maize FLRs share a common structure with AtFER with an amino‐terminal MLD and a carboxy‐terminal intracellular serine/threonine kinase domain (Figure S1a,b). The expression profile of the 14 maize putative CrRLK1Ls was analysed by using published maize GSE27004 data (PRJNA137659) (Sekhon et al., 2011). The mRNA expression patterns of *ZmFLR1* and *ZmFLR2* were very similar, with highest expression in silks and the pericarp, while *ZmFLR3* was expressed in all tissues except the embryo (Figure S2).

### 2.2 Subcellular localization of three homologous FLR family members

**FIGURE 2:**
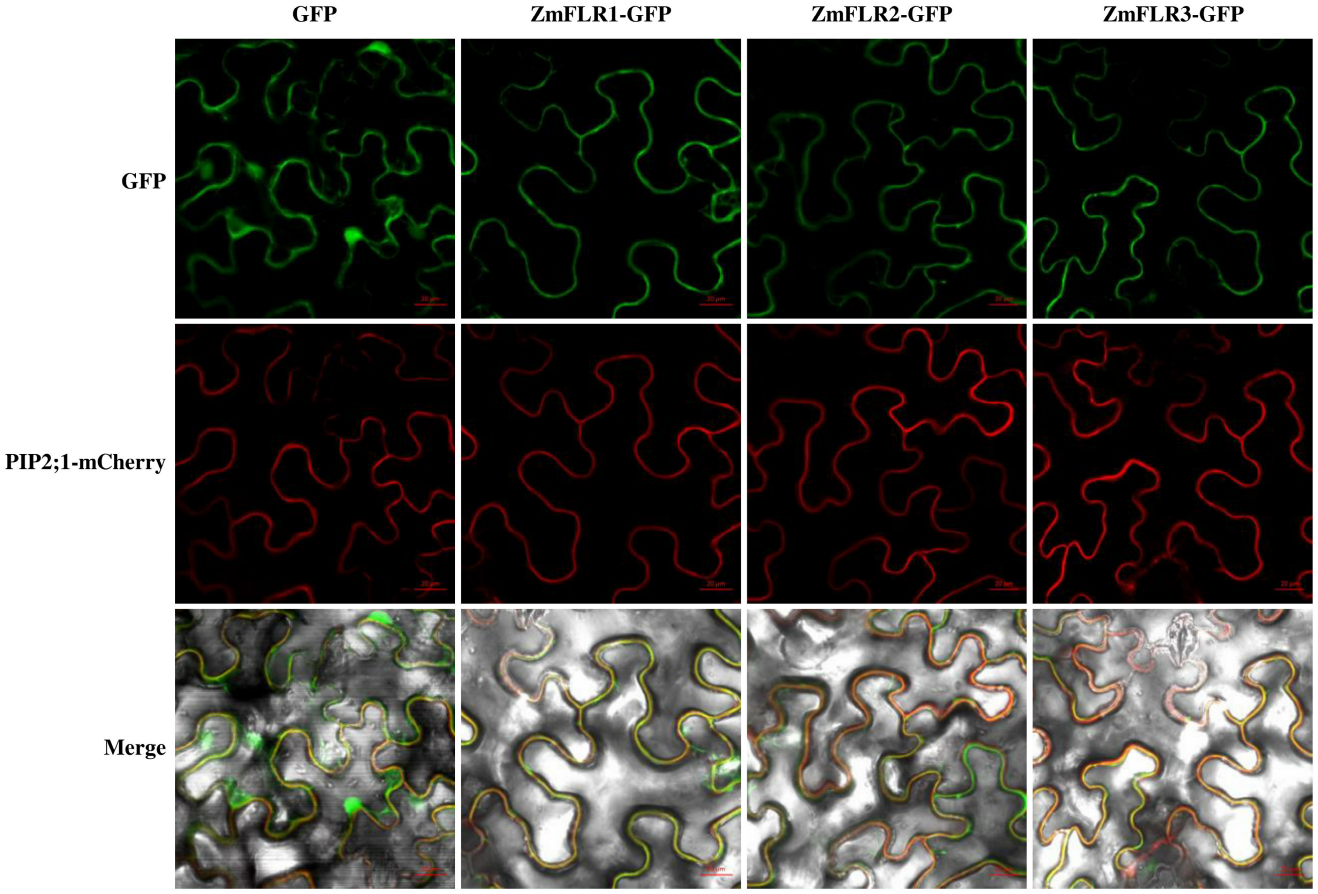
Subcellular localization of ZmFLRs in cell membrane. GFP, ZmFLRs‐GFP, and cell membrane marker of PIP2;1‐mCherry fusion proteins transformed into *Agrobacterium tumefaciens* and infiltrated into *Nicotiana benthamiana* leaves. Confocal microscopy images were taken at 36 h after infiltration. Scale bars = 20 μm.

Similar to *AtFER*, *ZmFLR1*, *ZmFLR2*, and *ZmFLR3* encode 888, 886, and 897 amino acid proteins, respectively, each containing the representative domains: the extracellular receptor domain (including the MLD), a transmembrane domain (TMD), and an intracellular serine/threonine kinase domain (Figure S1a). To examine the expression of ZmFLRs, transient expression assays of the ZmFLR‐green fluorescent protein (GFP) fusions were conducted in *Nicotiana benthamiana* leaves. The GFP control was observed in the cytoplasm and nucleus, and ZmFLRs‐GFP co‐located with the red fluorescence of protein PIP2;1‐mCherry (a cell membrane marker) (Lee et al., 2009), indicating that ZmFLRs have a plasma membrane localization (Figure 2).

### 2.3 ZmFLRs induce cell death in *N. benthamiana* leaves

**FIGURE 3:**
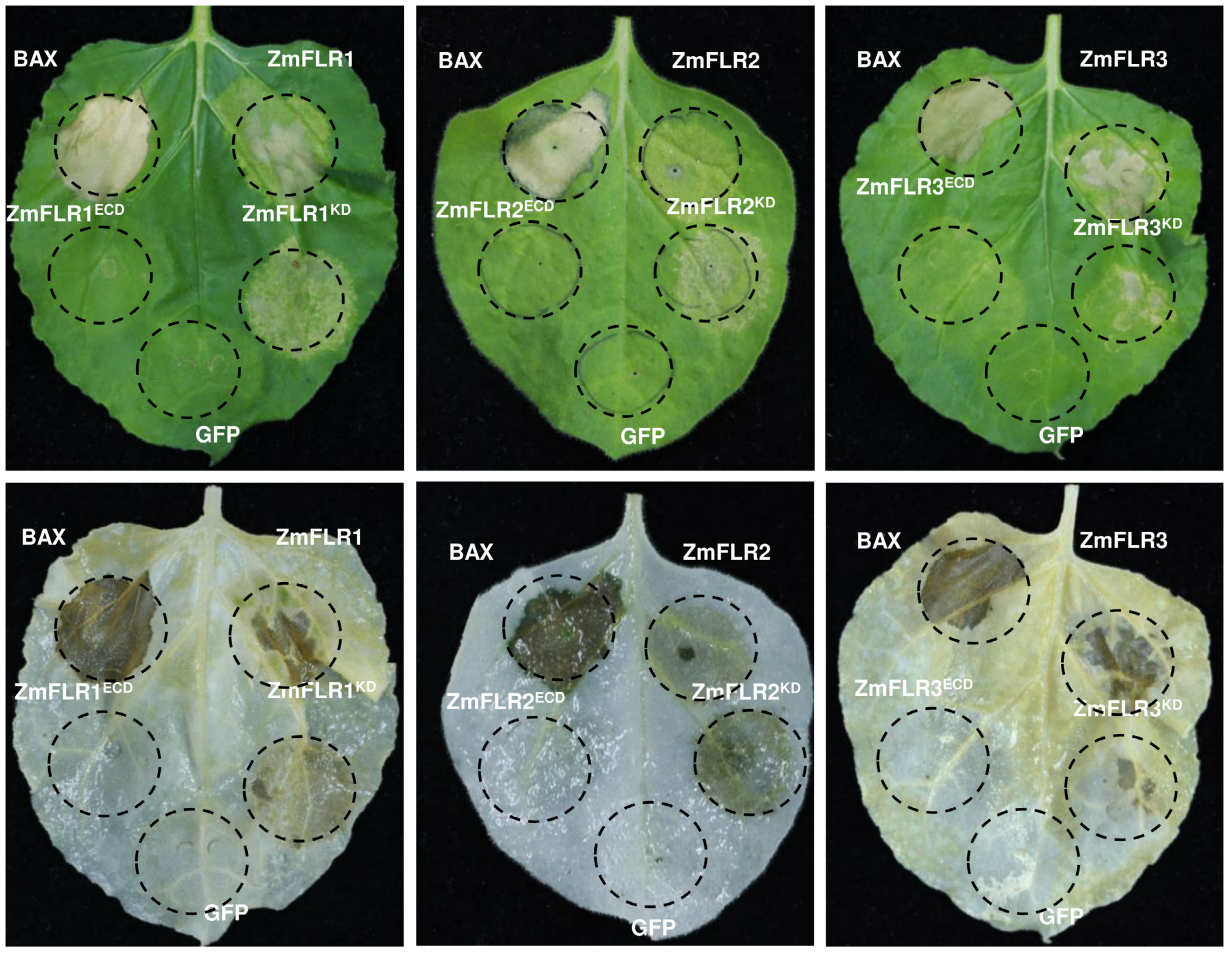
Transient expression of *ZmFLR*s‐induced cell death in *Nicotiana benthamiana* leaves. The cell death phenotype following infiltration of *Agrobacterium tumefaciens* cells containing *eGFP* (negative control), *BAX* (positive control), *ZmFLR*s, *ZmFLR*s^ECD^ or *ZmFLR*s^KD^. Photographs were taken 4 days after infiltration, and leaves were decolourized with ethanol. ECD, extracellular domain; KD, serine/threonine kinase domain.

To determine the function of ZmFLRs, we transiently overexpressed their coding sequences (CDSs), ECD, and serine/threonine kinase domain in *N. benthamiana*. We employed BAX as the positive control, which is able to trigger a strong cell death when expressed in tobacco (Lacomme & Santa Cruz, 1999). Four days after infiltration with *Agrobacterium* cells carrying *ZmFLR1*, *ZmFLR2*, or *ZmFLR3*, a strong cell death phenotype was observed in *N. benthamiana* leaves, and the kinase domain was responsible for the induction of cell death (Figure 3). We also coexpressed ZmFLRs with LUC in maize protoplast to detect cell death. We found that ZmFLR1 and ZmFLR2 induced cell death more rapidly than ZmFLR3 when incubated for 12 h (Figure S3). These results revealed that ZmFLRs may act as positive regulators of plant cell death.

### 2.4 The expression profile of *ZmFLR* genes in response to *S. turcica*, *B. zeicola*, *C. graminicola*, and *B. maydis*

**FIGURE 4:**
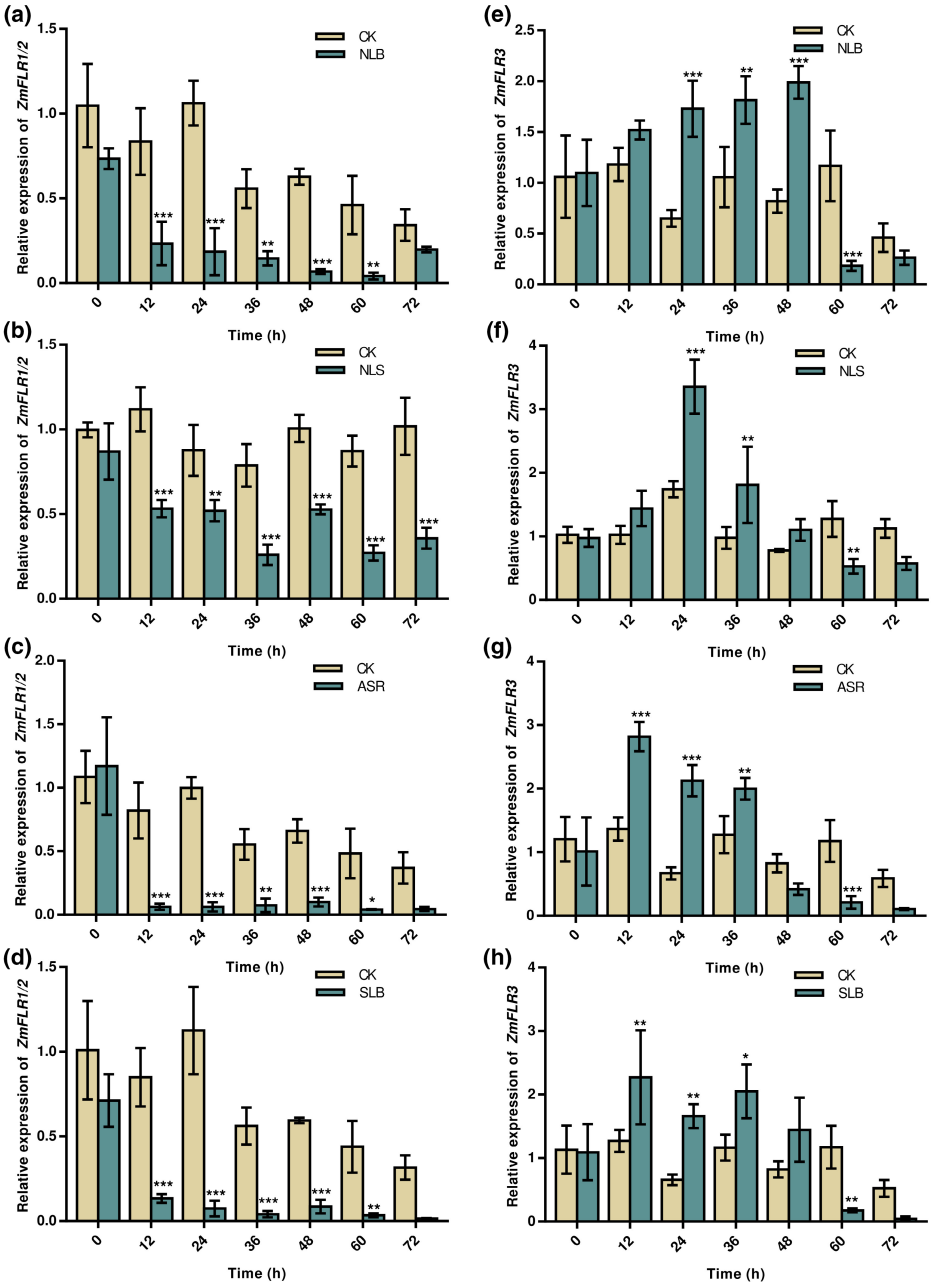
The expression patterns of *ZmFLR* genes in response to *Setosphaeria turcica* (northern corn leaf blight, NLB), *Bipolaris zeicola* (northern corn leaf spot, NLS), C*olletotrichum graminicola* (anthracnose stalk rot, ASR), and *Bipolaris maydis* (southern corn leaf blight, SLB). (a–d) The expression level of *ZmFLR1/2* against *S. turcica*, *B. zeicola*, *C. graminicola*, and *B. maydis* at the corresponding time. (e–h) The expression level of *ZmFLR3* against *S. turcica*, *B. zeicola*, *C. graminicola*, and *B. maydis* at the corresponding time. Values are mean ± *SD* (*n* = 3) and asterisks indicate significant differences (*p <* 0.05) using Student's *t* test between negative control (CK) and *S. turcica*, *B. zeicola*, *C. graminicola*, and *B. maydis* at each time point.

To investigate the role of *ZmFLR*s in response to various maize fungal diseases, the expression profiles of *ZmFLR1/2* and *ZmFLR3* genes were analysed in maize infected with *S. turcica*, *B. zeicol*a, *C. graminicola*, and *B. maydis*. The expression level of *ZmFLR1/2* was significantly down‐regulated at 12–72 h in response to *S. turcica*, *B. zeicola*, *C. graminicola*, and *B. maydis* compared to the control plants (Figure 4a–d). Unlike *ZmFLR1/2*, the transcript levels of *ZmFLR3* increased from 12 to 48 h and there were significant differences at time points 24, 36, and 48 h in plants inoculated with *S. turcica*, *B. zeicola*, *C. graminicola*, and *B. maydis* compared to the control plants (Figure 4e–h). However, the expression of *ZmFLR3* was significantly down‐regulated compared to the control at 60 and 72 h after pathogen inoculation.

### 2.5 *ZmFLR*s are required for the ROS burst triggered by flg22 and chitin

**FIGURE 5:**
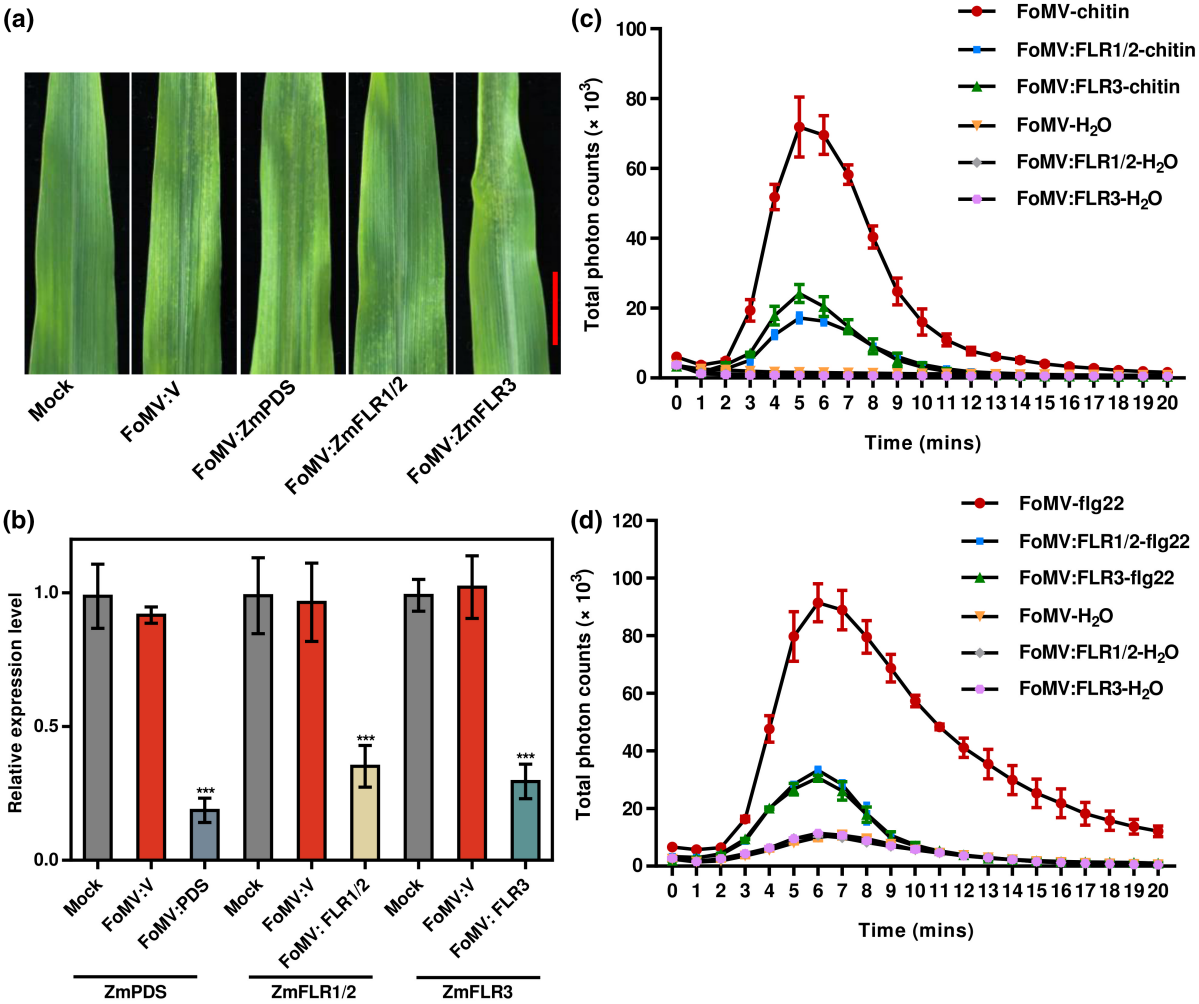
ZmFLRs are required for chitin‐ and flg22‐induced immunity. (a) Symptoms of foxtail mosaic virus (FoMV) infection and virus‐induced gene silencing (VIGS) phenotypes in *ZmFLR‐*silenced maize plants. (b) Reverse transcription‐quantitative PCR analysis of *ZmFLR* expression levels in noninfected (mock), FoMV‐V empty vector (FoMV:V), and FoMV‐ZmFLRs‐infected maize plants. Error bars indicate the *SD* of three technical replicates for each individual sample. Asterisks indicate significant differences (*p <* 0.05) using Student's *t* test. (c) Chitin‐induced reactive oxygen species (ROS) production in FoMV:FLRs and FoMV:V plants (mean ± *SD*, *n* = 3). (d) Flg22‐induced ROS production in FoMV:FLRs and FoMV:V plants (mean ± *SD*, *n* = 3).

Based on the cell death phenotype that ZmFLRs could trigger in *N. benthamiana* leaves and their differential expression following pathogen inoculation, we were interested in the function of *ZmFLR*s in maize immunity. *ZmFLR‐*silenced plants were generated via virus‐induced gene silencing (VIGS) mediated by foxtail mosaic virus (FoMV) (Beernink et al., 2021; Mei et al., 2016). Because of the striking photobleaching phenotype, the *ZmPDS* gene, encoding phytoene desaturase, was used as the positive control for the FoMV‐VIGS system. Because the *ZmFLR1* and *ZmFLR2* coding sequences are 97.2% identical, we silenced the two of them simultaneously to generate FoMV:FLR1/2 and FoMV:FLR3 plants. All FoMV‐inoculated B73 maize plants displayed mosaic symptoms at 7 days postinoculation (dpi) (Figure 5a). The *ZmFLR1/2* and *ZmFLR3* transcript levels were reduced by 66.2% and 70.7%, respectively (Figure 5b). At 14 days after FoMV infection, the fourth leaves of maize plants were used to explore the ROS burst triggered by chitin or flg22. The 4‐mm leaf discs were immersed in chitin or flg22 solution and the ROS signals were detected by applying a luminol chemiluminescence assay for 20 min. After chitin or flg22 treatment, a ROS burst peaked at 4 or 6 min. FoMV:FLR1/2‐ and FoMV:FLR3‐silenced plants both showed a reduced ROS burst following treatment with flg22 or chitin compared to FoMV:V plants (Figure 5c,d). These results showed that *ZmFLR*s positively regulate immunity in maize.

### 2.6 ZmFLRs confer resistance to multiple pathogens

**FIGURE 6:**
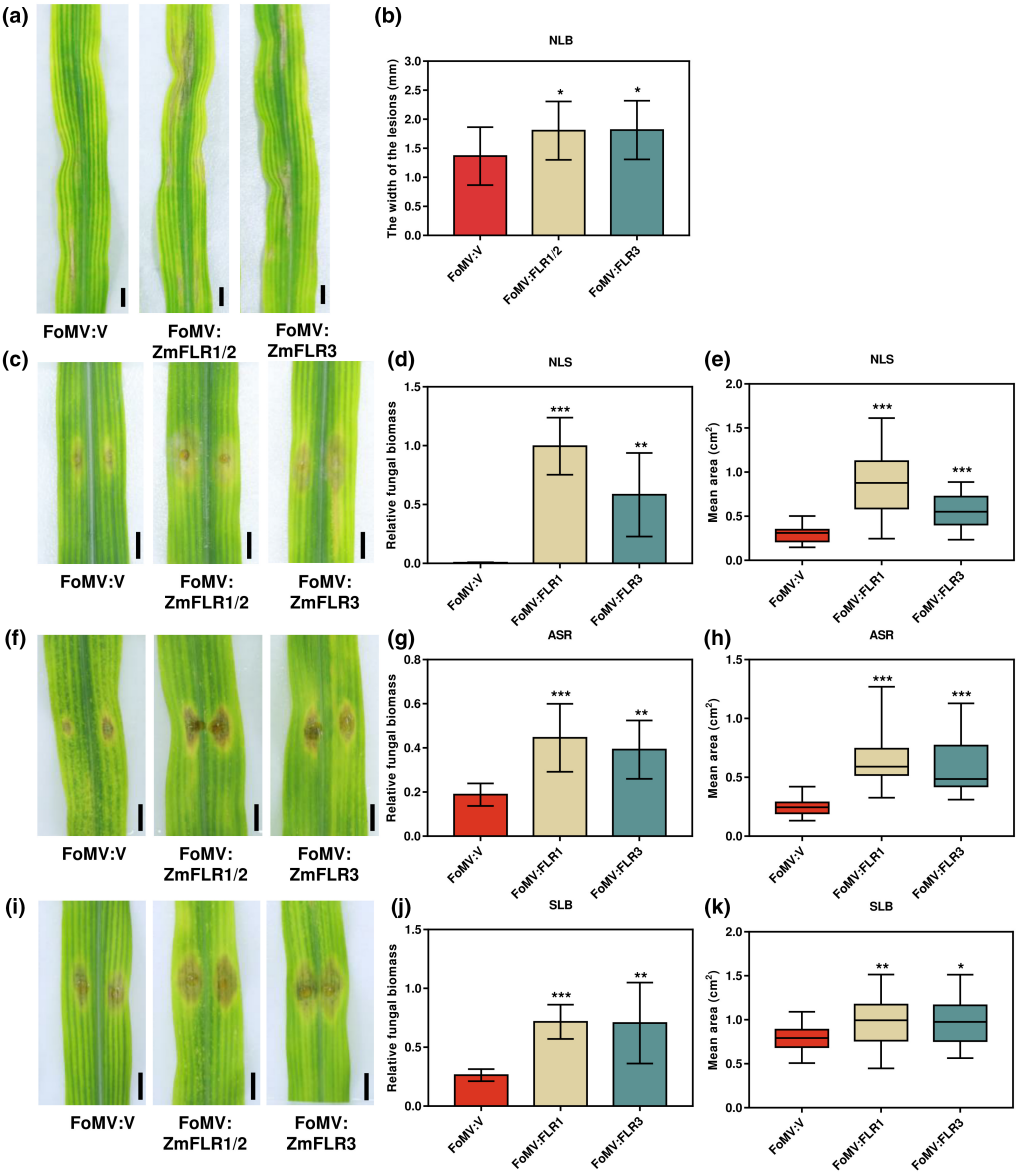
FoMV‐mediated virus‐induced gene silencing (VIGS) of *ZmFLR*s increased susceptibility of maize plants to *Setosphaeria turcica* (northern corn leaf blight, NLB), *Bipolaris zeicola* (northern corn leaf spot, NLS), *Colletotrichum graminicola* (anthracnose stalk rot, ASR), and *Bipolaris maydis* (southern corn leaf blight, SLB). (a) The disease phenotypes of FoMV:V, FoMV:FLR1/2, and FoMV:FLR3 plants against *S. turcica*. (b) The width of the *S. turcica* lesions on FoMV:FLR1/2, FoMV:FLR3, and FoMV:V plants. Thirty‐two lesions on FoMV:V plants, 23 lesions on FoMV:FLR1/2, and 21 lesions on FoMV:FLR3 plants were analysed. (c) The disease phenotypes of FoMV:V, FoMV:FLR1/2, and FoMV:FLR3 plants incoulated with *B. zeicola*. (d) Relative fungal biomass of *B. zeicola* in FoMV:V, FoMV:FLR1/2, and FoMV:FLR3 plants was measured using quantitative PCR (qPCR) by calculating 2^[*C*t(*ZmActin*) − *C*t(*Tubulin*)]^. (e) The lesion area of *B. zeicola* on FoMV:V, FoMV:FLR1/2, and FoMV:FLR3 plants was measured using ImageJ. (f) The disease phenotypes of FoMV:V, FoMV:FLR1/2, and FoMV:FLR3 plants inoculated with *C. graminicola*. (g) Relative fungal biomass of *C. graminicola* in FoMV:V, FoMV:FLR1/2, and FoMV:FLR3 plants was measured using qPCR by calculating 2^[*C*t(*ZmActin*) − *C*t(*Tubulin*)]^. (h) The lesion area of *C. graminicola* on FoMV:V, FoMV:FLR1/2, and FoMV:FLR3 plants was measured using ImageJ. (i) The disease phenotypes of FoMV:V, FoMV:FLR1/2, and FoMV:FLR3 plants inoculated with *B. maydis*. (j) Relative fungal biomass of *B. maydis* in FoMV:V, FoMV:FLR1/2, and FoMV:FLR3 plants was measured using qPCR by calculating 2^[*C*t(*ZmActin*) − *C*t(*Tubulin*)]^, total DNA was extracted from control FoMV:V and *ZmFLR*‐silenced maize plants. Data are shown as mean ± *SE* (*n* = 3) and asterisks indicate significant differences (*p <* 0.05) using Student's *t* test. (k) The lesion area of *B. maydis* on FoMV:V, FoMV:FLR1/2, and FoMV:FLR3 plants was measured using ImageJ. Data are shown as mean ± *SE* (*n* = 6) and asterisks indicate significant differences (*p <* 0.05) using Student's *t* test.

To further investigate the character of *ZmFLR*s in the resistance to major foliar fungal diseases in maize, we assessed the resistance of the FoMV:FLR1/2‐ and FoMV:FLR3‐silenced plants to *S. turcica*, *B. zeicola*, *C. graminicola*, and *B. maydis*. The FoMV:FLR1/2‐ and FoMV:FLR3‐silenced plants showed more susceptibility to *S. turcica*, *B. zeicola*, *C. graminicola*, and B. maydis than the FoMV:V plants (Figure 6a,c,f,i). Notably, the lesion width of *S. turcica* on FoMV:FLR1/2‐ and FoMV:FLR3‐silenced plants was significantly wider than those on FoMV:V plants (Figure 6b). Furthermore, the lesion area and relative fungal biomass of *B. zeicola*, *C. graminicola*, or *B. maydis* were significantly higher than those of FoMV:V control plants (Figure 6d,e,g,h,j,k). These results strongly suggest that *ZmFLR*s positively regulate resistance against *S. turcica*, *B. zeicola*, *C. graminicola*, and *B. maydis*.

**FIGURE 7:**
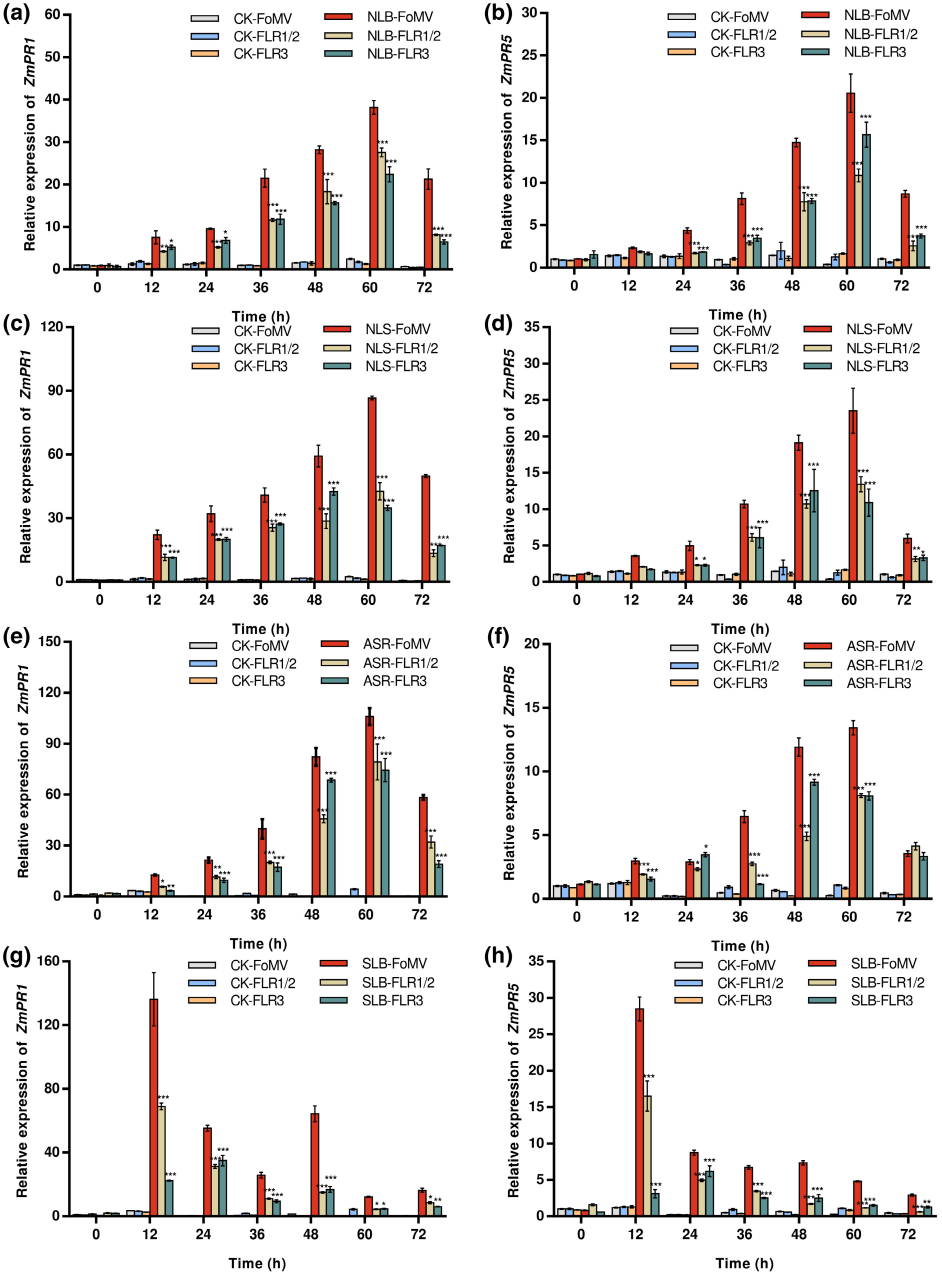
The expression patterns of *ZmPR1* and *ZmPR5* in FoMV:V, FoMV:FLR1/2, and FoMV:FLR3 silenced plants in response to *Setosphaeria turcica* (northern corn leaf blight, NLB), *Bipolaris zeicola* (northern corn leaf spot, NLS), *Colletotrichum graminicola* (anthracnose stalk rot, ASR), and *Bipolaris maydis* (southern corn leaf blight, SLB). (a–d) The expression level of *ZmPR1* after inoculation with *S. turcica*, *B. zeicola*, *C. graminicola*, and *B. maydis* over time. (e–h) The expression level of *ZmPR5* after inoculation with *S. turcica*, *B. zeicola*, *C. graminicola*, and *B. maydis* over time. Values are mean ± *SD* (*n* = 3) and different letters indicate significant differences (*p* < 0.05) among the treatments at the same time. Asterisks indicate significant differences using Dunnett's test (*p <* 0.05) between FoMV and FLR1/2, FLR3 silenced plants in response to the four pathogens at each time point.

To investigate the role of *ZmFLR*s in maize defence against *S. turcica*, *B. zeicola*, *C. graminicola*, and *B. maydis*, we evaluated the expression of maize immune response marker genes, pathogenesis‐related genes *ZmPR1*, *ZmPR5* (Kong & Li, 2011), *ZmPR3*, and *ZmPR4* (Ziemann et al., 2018). The expression level of *ZmPR1* and *ZmPR5* in FoMV:V and FoMV:*ZmFLR*s plants under control (noninfected) conditions showed no significant difference. *S. turcica* inoculation caused a rapid increase in expression of *ZmPR1* and *ZmPR5* in both FoMV:V and FoMV:*ZmFLR*s plants, which peaked at 60 h and then decreased thereafter (Figure 7a). *ZmFLR‐*silenced maize plants inoculated with *S. turcica* had significantly reduced expression levels of *ZmPR1* and *ZmPR5* relative to the FoMV:V control plants (Figure 7b). Similar expression patterns of *ZmPR1* and *ZmPR5* were observed in response to *B. zeicola* (Figure 7c,d) and *C. graminicola* (Figure 7e,f). Unlike the hemibiotrophic *S. turcica*, *B. zeicola*, and *C. graminicola*, the necrotrophic *B. maydis* also sharply induced the expression of *ZmPR1* and *ZmPR5* in both FoMV:V and FoMV:ZmFLRs plants, but expression reached a peak at 12 h and then decreased (Figure 7g,h). The expression levels of *ZmPR3* and *ZmPR4* were similar to *ZmPR1* and *ZmPR5* in response to all four pathogens (Figure S4).

## 3 DISCUSSION

The members of the *Cr*RLK1L subfamily exist specifically and extensively in plants and, similar to all RLK family members, they possess three typical domains (extracellular, transmembrane, and a relatively conserved serine/threonine kinase domain) (Boisson‐Dernier et al., 2011; Schallus et al., 2008). FER homologues can be found in lower plants such as *M. polymorpha* and *C. richardii* as well as in higher plants such as tomato, cotton, cinnamon, and poplar (Figure 1). The extracellular domain of *Cr*RLK1Ls is dissimilar to the extracellular domain of RLKs (Takeda et al., 2014). *Cr*RLK1 was first discovered to have Mn^2+^‐dependent serine/threonine protein kinase activity but, unlike other RLKs members, the kinase activity of *Cr*RLK1 is achieved through intramolecular rather than intermolecular autophosphorylation (Schulze‐Muth et al., 1996). The serine/threonine kinase domain of FER has been shown to have autophosphorylation activity in an *in vitro* kinase assay (Kessler et al., 2015).

Although *Cr*RLK1L is a small receptor‐like protein kinase subfamily in plants, it has been shown to be extensively expressed in different plant tissues (Franck et al., 2018). Increasing evidence indicates that *Cr*RLK1L members probably use varied motifs to interact with diverse ligands or signal molecules in different tissues, organs, or developmental stages (Franck et al., 2018). Furthermore, due to the difference in the number or activity of ligands or signal molecules, different signal transduction mechanisms are initiated, leading to different biological effects (Boisson‐Dernier et al., 2013; Fujikura et al., 2014; Guo et al., 2009; Hématy et al., 2007). Analysis of the expression patterns of 14 *ZmFLR*s revealed that they are expressed in different vegetative and reproductive organs. The expression patterns of *ZmFLR1* and *ZmFLR2* were very similar and highly expressed in silks and the pericarp, while *ZmFLR3* was highly expressed in all tissues except embryos (Figure S2). This differential expression indicates that there is subfunctionalization and that *ZmFLR1* and *ZmFLR2* may work in a different manner to *ZmFLR3* in regulating seed size.

PTI protects plants in a broad manner in response to most pathogens without a growth penalty (Yang et al., 2020). On perception of PAMPs by PRRs, immune signalling responses such as ROS production, defence‐related gene activation, and MAPK cascades are immediately initiated to promote defence responses (Boller & Felix, 2009). The FER kinase domain can directly interact with Rop‐guanine exchange factors (ROPGEFs), which further interact with NADPH oxidase to regulate ROS levels and ultimately plant immunity (Li et al., 2015; Nagano et al., 2016; Nibau & Cheung, 2011). FER also positively regulates flg22‐induced ROS accumulation during immune responses, whereas it negatively regulates ROS levels in guard cells associated with ABA (Yu et al., 2012). In rice, two *OsFLR* mutants, *flr2* and *flr11*, *M. oryzae* infection caused both increased expression of defence‐related gene and accumulation of ROS (Yang et al., 2020). In soybean, the *Gmlmm1* mutant shows significantly enhanced ROS production after flg22 or chitin treatment, and *GmLMM1* strongly suppresses XEG1‐induced cell death, suggesting that GmLMM1 negatively regulates PTI responses (Wang et al., 2020). It is not common that cell surface receptors alone induce cell death. SOBIR1 (suppressor of *bir1‐1*) encodes a receptor‐like kinase; the *sobir1‐1* mutation suppresses cell death in *bir1‐1*, while overexpression of *SOBIR1* in both *Arabidopsis* and *N. benthamiana* is highly phosphorylated and activates cell death and defence responses (van der Burgh et al., 2019; Gao et al., 2009). This indicates that *SOBIR1* plays a positive role in initiating the immune response. In addition, SOBIR1 transphosphorylates BAK1 and the activated receptor complex works to induce downstream defence signalling (van der Burgh et al., 2019). Further functional analysis verified that the kinase domain of SOBIR1 is response for induction of cell death in *N. benthamiana* (Wei et al., 2022). It has been shown that FERONIA receptor kinases can regulate ROS production and function as a scaffold protein for PAMP receptors, playing a positive role in PTI and plant immunity (Stegmann et al., 2017). In the present study, overexpression of *ZmFLR*s in *N. benthamiana* leaves induced cell death and the kinase domain was responsible for the hypersensitive response (Figure 3). FoMV:FLR1/2‐ and FoMV:FLR3‐silenced plants both showed a reduced ROS burst after flg22 or chitin treatment compared with FoMV:V plants (Figure 5c,d). Taken together, our data suggest that *ZmFLR*s may positively regulate PTI. This conclusion is consistent with the observation that AtFER probably serves as a scaffold protein to promote the ligand‐induced FLS2‐BAK1 and ERF‐BAK1 interactions (Stegmann et al., 2017). The other two members of the *Cr*RLK1L family, ANX1 and ANX2, interact with FLS2 to negatively regulate FLS2‐mediated antibacterial immunity, possibly by inducing segregation of BAK1 (Mang et al., 2017). GmLMM1 can restrain the FLS2‐BAK1 sequestration with flg22 treatment, and it serves as a molecular adjustor in regulating immune activation by controlling the FLS2‐BAK1 interaction (Wang et al., 2020). Therefore, different scaffold proteins could be recruited by a single PRR to either positively or negatively regulate its function. We speculate that ZmFLRs may bind to BAK1 to induce downstream defence signalling. PR proteins, which are downstream of FER genes, can induce plant programmed cell death, which inhibits the spread of infection. We hypothesize that overexpression of ZmFLRs subsequently induces the activation and overexpression of PR proteins, leading to programmed cell death. In addition, the reduction in PR gene expression in *ZmFLR*‐silenced plants suggests that ZmFLRs act upstream of these immune‐related genes. PR proteins are functionally diverse proteins that are inducible during a pathogen attack and are regulated by signalling compounds such as ABA, ethylene, jasmonic acid, and salicylic acid. Therefore, defence against different classes of pathogens can be mediated by PR proteins (Loake & Grant, 2007; Van Loon et al., 2006).

When challenged with fungal pathogens, *fer* mutant plants were more resistant to *G. orontii*, *F. oxysporum*, and *M. oryzae* (Kessler et al., 2010; Masachis et al., 2016). Similarly, mutants of the FER homologous genes *Osflr2*, *Osflr11*, and *Gmlmm1* also have enhanced resistance to *M. oryzae* and oomycete pathogens (Wang et al., 2020; Yang et al., 2020). Nevertheless, this research does not sufficiently indicate that FER negatively regulates immunity in this circumstance, but rather that FER and its dependent signalling pathways are frequently targeted by pathogenic fungi (Franck et al., 2018). In our current study, *ZmFLR*s conferred enhanced resistance to *S. turcica*, *B. zeicola*, *C. graminicola*, and *B. maydis*. The FoMV:FLR1/2‐ and FoMV:FLR3‐silenced plants were significantly more susceptible to these four pathogens than the FoMV:V plants (Figure 6). These results indicate that *ZmFLR*s may positively regulate resistance against *S. turcica*, *B. zeicola*, *C. graminicola*, and *B. maydis*.

NLB, NLS, ASR, and SLB are the main maize foliar fungal diseases worldwide (Balint‐Kurti & Johal, 2009). NLB, NLS, and ASR are caused by the hemibiotrophic fungi *S. turcica*, *B. zeicola*, *C. graminicola*, respectively. These pathogens use hemibiotrophic infection strategies with multiple steps. First, a dome‐shaped appressorium penetrates the host surface through mechanical pressure and enzymatic hydrolysis to form biotrophic hyphae, which inhibit plant immunity and obtain nutrients from living cells. Later, these fungi switch to a necrotrophic phase in which rapidly growing hyphae kill and destroy host tissues (Kleemann et al., 2012; Liu et al., 2015; Wang et al., 2021). SLB is caused by the necrotrophic fungus *B. maydis*. Necrotrophs are plant pathogens that degrade plant components or kill the plant by secreting lytic enzymes or toxins. Subsequently, the pathogen acquires nutrients from dead or dying tissues (Mayer et al., 2001; Shao et al., 2021). The maize pathogen *Cochliobolus heterostrophus* secretes a DNase, NUC1, which acts as a virulence factor for defence against host‐secreted extracellular DNA (Park et al., 2019). Another transcription repressor, ZmMM1, can positively regulate plant immune responses and confers broad‐spectrum disease resistance to *S. turcica* (hemibiotrophic fungus), *Cercospora zeae‐maydis* (necrotrophic fungus), and *Puccinia polysora* (biotrophic fungus) (Wang et al., 2021). In the present study, the pathogens achieved infection by inhibiting the expression of *ZmFLRs* in maize B73 with normal expression levels (Figure 4). The necrotrophic fungus *B. maydis* rapidly induced the expression of *ZmPR1* and *ZmPR5* compared to the hemibiotrophic fungi *S. turcica*, *B. zeicola*, and *C. graminicola*, reaching a peak at 12 h (Figure 7). In addition, when LUC was coexpressed with ZmFLR1, ZmFLR2, and ZmFLR3 in maize protoplasts, ZmFLR1 and ZmFLR2 induced cell death more rapidly than ZmFLR3 (Figure S3). We speculate when plants suffer from pathogen attack, ZmFLR1 and ZmFLR2 sharply induce cell death, causing a strong immune response. In order to maintain their physiological and biochemical activities, plants inhibit the transcriptional expression of *ZmFLR1/2*. There may be a feedback regulation in response to pathogenic infection.

In summary, this study demonstrates that the maize homologues of the *Cr*RLK1L subfamily member AtFER, ZmFLRs, harbour the typical ECD, transmembrane domain, and serin/threonine kinase domain, and are localized to the cell membrane. We showed that overexpression of ZmFLRs in *N. benthamiana* leaves induced plant cell death. In addition, FoMV:FLR1/2‐ and FoMV:FLR3‐silenced plants showed a reduced ROS burst after treatment with the PAMPs chitin or flg22. ZmFLRs positively regulated resistance to *S. turcica*, *B. zeicola*, *C. graminicola*, and *B. maydis*. The FoMV:FLR1/2‐ and FoMV:FLR3‐silenced plants were more sensitive to the four pathogens than the FoMV:V plants. These results indicate that ZmFLRs may positively regulate PTI. Thus, *ZmFLR*s are positively involved in broad‐spectrum disease resistance in maize.

## 4 EXPERIMENTAL PROCEDURES

### 4.1 Identification of *FLR* genes in maize

The CDS and protein sequence data of maize B73 (*Z. mays*) were downloaded from the Maize Genetics and Genomics Database (Maize GDB: https://maizegdb.org/). Sixteen FLRs of *Oryza sativa* japonica rice (Yang et al., 2020) were downloaded from the Rice Genome Annotation Project database (RGAP, http://rice.plantbiology.msu.edu/). *Arabidopsis* FERONIA protein sequences (Lindner et al., 2012) were downloaded from TAIR (https://www.arabidopsis.org/). The potential FLR genes in maize and 31 FER homologues from different plant families were identified by the BLAST in Phytozome (https://phytozome.jgi.doe.gov/pz/portal.html) using *AtFER* as the query. The Simple Modular Architecture Research Tool (SMART, http://smart.embl‐heidelberg.de/) was used to confirm the candidate sequences that contained both conserved domains.

The expression profiles of 14 maize *Cr*RLK1L family members in different tissues were analysed using published maize GSE27004 data (PRJNA137659) (Sekhon et al., 2011). The expression data of the 14 maize *Cr*RLK1L genes were extracted from the total expression data by internal Perl script and the heatmap was drawn with R packages (pheatmap v. 4.1.1).

### 4.2 Phylogenetic tree construction and domain organization analysis

All sequences of *Cr*RLK1L proteins from *Arabidopsis*, maize, and rice were aligned using ClustalW software with the default parameters (http://www.clustal.org/clustal2/). Subsequently, MEGA 7 was used to construct a rooted, neighbour‐joining method phylogenetic tree and calculate the genetic distance for the *Cr*RLK1L protein sequences. The parameters were set as follows: 1000 bootstrap replications and all positions containing gaps and missing data were deleted. The phylogenetic tree was optimized using iTOL (https://itol.embl.de/). Protein domain structure visualization was constructed using DOG 2.0 (http://dog.biocuckoo.org/).

### 4.3 Plant materials, fungal strains, and growth conditions

Maize B73 inbred line (wild type) and *N. benthamiana* were used in this research. Maize seeds were sown in a pot (10 × 8 cm deep) containing a mixture of vermiculite and commercial garden soil (1:3; vol/vol) and were grown in a greenhouse with a 14‐h photoperiod, a temperature cycle of 24°C/20°C day/night, 300 mmol⋅m^−2^ ⋅s^−1^ irradiance, and relative humidity of 50%–60%. *N. benthamiana* seeds were surface‐sterilized, germinated in 1/2 × Murashige‐Skoog (MS) medium plate for 6 days, and transplanted to the same soil and growth conditions as maize. The *N. benthamiana* plants were used for agroinfiltration and subcellular localization observation. The following pathogen strains were used: *S. turcica* (strain 21–2‐1, isolated from Gongzhuling, Jilin province), *B. maydis* (strain 4–4‐3, isolated from Wudalianchi, Heilongjiang province), *C. graminicola* (strain CgM2), and *B. zeicola* (strain 7–1‐2, isolated from Wudalianchi, Heilongjiang province). All strains were cultured on oatmeal agar plates and incubated at 25°C in the dark for 1 week, then placed under a 12‐h photoperiod at 25°C until sporulation.

To determine the transcript levels of *ZmFLR1/2* and *ZmFLR3* during *S. turcica, B. zeicola, C. graminicola*, and *B. maydis* infection, we sprayed suspensions of 10^5^ spores/ml on the 14‐day‐old seedlings and samples were taken at 0, 12, 24, 36, 48, 60, and 72 h postinoculation (hpi). The expression levels at 0 hpi with water treatment were used as calibrator samples. Reverse transcription‐quantitative PCR (RT‐qPCR; primers in Table S1) was used to assay the transcript levels of *ZmFLR1/2* and *ZmFLR3*.

### 4.4 Construction of *Agrobacterium*‐mediated maize VIGS plants and plant inoculation

VIGS on maize plants was carried out according to the previous method (Beernink et al., 2021) with minor modifications. The coding sequences of *ZmFLR1* and *ZmFLR2* are 2667 and 2694 bp in length, respectively, encoding predicted proteins with 97.5% similarity. It is virtually impossible to silence these two genes separately. The serin‐threonine kinase domain and MLDs of these three genes are quite conserved, therefore the transmembrane domain was selected to design the VIGS primers. We cloned 279 bp of *ZmFLR1/2* and 210 bp of *ZmFLR3* from maize cDNA into FoMV‐pCAMBIA1380 binary vectors in the antisense orientation. FoMV:PDS carrying the maize phytoene desaturase (*PDS*) gene and FoMV:V were used as controls for the FoMV infection assay. Then we introduced these plasmid constructs into *Agrobacterium tumefaciens* GV3101 using the freeze–thaw method. For the infiltration, the *Agrobacterium* cells were pelleted and resuspended in infiltration buffer (10 mM MgSO_4_, 200 μM acetosyringone) to an OD_600_ of 1.0. The *Agrobacterium* suspension was injected 2–3 mm above the coleoptilar node of 5‐day‐old seedlings. Plants were grown for another 14 days after injection to observe symptoms. The silencing efficiency of *ZmFLR1/2* or *ZmFLR3* was validated using RT‐qPCR from the middle part of the fourth leaf (Livak & Schmittgen, 2001). Plants were cultivated for another week and the fourth to sixth leaves with viral symptoms were harvested in a 50‐ml tube with drierite desiccant in the bottom, lyophilized overnight to dry completely, and stored at −20°C. Rub inoculation is simple and has a nearly 90% infection rate, making it easy to generate gene‐silenced plants. Approximately 100 mg of lyophilized tissue was ground in 50 mM potassium phosphate buffer (pH 7.0). Maize leaves were dusted with carborundum and the leaf sap solution. Rub‐inoculation was performed using a gloved finger to rub the drop of inoculum over the leaf surface. Next, inoculated leaves were rinsed with tap water to remove excess carborundum. Inoculated plants were then placed in the greenhouse for approximately 14 days to observe symptoms. At this time, plants were ready for the next in vitro or spray inoculation with *S. turcica*, *B. zeicola*, *C. graminicola*, and *B. maydis*.

### 4.5 Pathogen inoculation assay

For *S. turcica*, the spore suspension was sprayed onto the maize VIGS plants at a concentration of 10^5^ spores/ml and samples were taken at 0, 12, 24, 36, 48, 60, and 72 hpi to detect the PR‐protein gene expression. The lesion width was measured at 10 dpi inoculation with ImageJ software. The average lesion width was calculated from at least 20 randomly selected lesions (Wang et al., 2021). For *B. zeicola*, *C. graminicola*, and *B. maydis*, the spore suspensions were sprayed on the maize VIGS plants at a concentration of 10^5^ spores/ml and samples were taken at 0, 12, 24, 36, 48, 60, and 72 hpi to detect the PR‐protein gene expression. For the pathogen quantification, the fourth maize leaf was detached, placed in a petri dish (25 × 25 cm) containing wet filter paper, and inoculated with a spore suspension of 10^5^ spores/ml. Inoculated leaves were cultured in a chamber at 95% humidity. Leaves were sampled at 5 dpi from the fourth leaf with about the same area. All primers used for VIGS plasmid construction and pathogen quantification are listed in Table S1. Photographs of diseased maize leaves were taken and the lesion areas were calculated by using ImageJ.

### 4.6 RNA isolation, gene expression, and pathogen quantification analysis

Total RNA was extracted using a FOREGENE Plant Total RNA Isolation Kit according to the manufacturer's instructions. Approximately 1 μg of total RNA was reverse transcribed using HiScript II Q RT SuperMix for qPCR (+gDNA wiper) (Vazyme). For pathogen quantification analysis, plant and fungal DNA were extracted with the CTAB method described by Zhang et al. (2010). A real‐time qPCR assay was performed on a 7500 real‐time PCR system (Applied Biosystems) using a RealStar Green Fast Mixture kit (Genestar).

### 4.7 ROS production assay

At 14 days after FoMV inoculation, a minimum of 30 leaf discs was taken from the five plants with a 4‐mm diameter puncher. The leaf discs were incubated in 20 ml of sterile water on a 9‐cm petri dish overnight in darkness. Then the leaf discs were transferred to 1.5‐ml tubes containing 100 μl of luminol (Bio‐Rad Immun‐Star horseradish peroxidase substrate), 1 μl of horseradish peroxidase (HRP), and 1 μl of 1 mM flg22 or 1 μl of 0.8 mM chitin. The signal was then immediately collected using a Glomax20/20luminometer (Promega) every minute for a total of 20 min. Three biological replicates were assayed for each sample.

### 4.8 Transient expression of *ZmFLR*s in *N. benthamiana*

*ZmFLR1*, *ZmFLR2*, and *ZmFLR3* were cloned into the pCAMBIA1300‐GFP vector and then these plasmid constructs were introduced into *A. tumefaciens* EHA105 using the electroporation method. For subcellular localization of *ZmFLR1*, *ZmFLR2*, or *ZmFLR3* in *N. benthamiana* leaves, the cells were harvested and resuspended in an infiltration buffer (10 mM MES pH 5.6, 10 mM MgCl_2_, 200 μM acetosyringone) to an OD_600_ of 1.0. The suspensions were infiltrated into 6‐week‐old *N. benthamiana* leaves (Lee et al., 2009). At 2 days after infiltration, the fluorescence was detected with a confocal microscope (LSM 980; Zeiss).

For *ZmFLR*s‐induced cell death, *A. tumefaciens* cells carrying *BAX* and *ZmFLR*s were collected and resuspended to a final OD_600_ of 0.2 and 1.0 with infiltration buffer, respectively. *ZmFLR*s, *ZmFLR*s^ECD^ or *ZmFLR*s^KD^ and *BAX* were infiltrated into the same *N. benthamiana* leaves. *A. tumefaciens* cells carrying *eGFP* were infiltrated as a negative control. The cell death phenotypes were analysed 4 days after transient expression. The leaves were cleared in boiling ethanol for 10 min until the chlorophyll was completely removed and then photographed. Each assay had at least three biological replicates.

### 4.9 Transient expression of *ZmFLR*s in maize protoplast

Maize protoplasts were isolated from 10‐day‐old etiolated seedlings according to the method described previously (Yu et al., 2021). Then 5 μg of pCAMBIA1300‐GFP‐ZmFLRs and pRTV‐myc‐LUC was coexpressed in 250 μl of maize protoplasts. After 12 h of incubation in the dark at room temperature, 1 mM D‐luceferin (Biovison) was mixed with the resuspended protoplasts and the luminescence signal from each sample was collected using a GloMax 96 microplate luminometer (Promega).

### 4.10 Statistics analysis

The data were statistically analysed using Prism v. 7.00 (GraphPad Software Inc.). Dunnett's test was calculated for multiple comparisons, and Student's unpaired *t* test was used for pairwise comparisons. *p* values <0.05 were considered significant.

## Supporting information


**Figure S1** The functional domain organization of the ZmFLR proteins. (a) Schematic representation of ZmFLRs and AtFER with their domain organization. (b) Sequence alignment between ZmFLR1, ZmFLR2, ZmFLR3, and AtFER. Red rectangles, malectin‐like domain; green rectangles, kinase domain

**Figure S2** Expression patterns of maize *Cr*RLK1L family genes in different organs (*n* = 3 for each group)

**Figure S3** Transient expression of *ZmFLR*s induced cell death in maize protoplasts. The luciferase activity was detected in maize protoplasts that coexpressed ZmFLRs with LUC. GFP was treated as a control and data were collected after 12 h of incubation. Values are mean ± *SD* (*n* = 8), and different letters indicate significant differences (*p* < 0.05) among the treatments at the same time using Student’s *t* test

**Figure S4** The expression patterns of *ZmPR3* and *ZmPR4* in FoMV:V, FoMV:FLR1/2, and FoMV:FLR3 silenced plants in response to *Setosphaeria turcica* (northern corn leaf blight, NLB), *Bipolaris zeicola* (northern corn leaf spot, NLS), *Colletotrichum graminicola* (anthracnose stalk rot, ASR), and *Bipolaris maydis* (southern corn leaf blight, SLB). (a–d) The expression level of *ZmPR3* against the four pathogens over time. (e–h) The expression level of *ZmPR4* against the four pathogens over time. Values are mean ± *SD* (*n* = 3) and asterisks indicate significant differences using Dunnett’s test (*p <* 0.05) between FoMV and FLR1/2, FLR3 silenced plants in response to the four pathogens at each time point

**Table S1** Primers used in this study

**Table S2** Genetic distance in 62 *Cr*RLK1Ls

## Data Availability

The sequences are available at GenBank (https://www.ncbi.nlm.nih.gov/genbank/ as accession numbers ZmFLR1: AQL06862; ZmFLR2: ONL97729; ZmFLR3: ONM13343. Other data that support the finding of this study are available from the corresponding author upon reasonable request.
