# Supplementary figures and images for: Maize FERONIA‐like receptor genes are involved in the response of multiple disease resistance in maize
Source: Mol Plant Pathol. 2022 May 21;23(9):1331–45. doi: 10.1111/mpp.13232 (PMC9366073; doi:10.1111/mpp.13232)

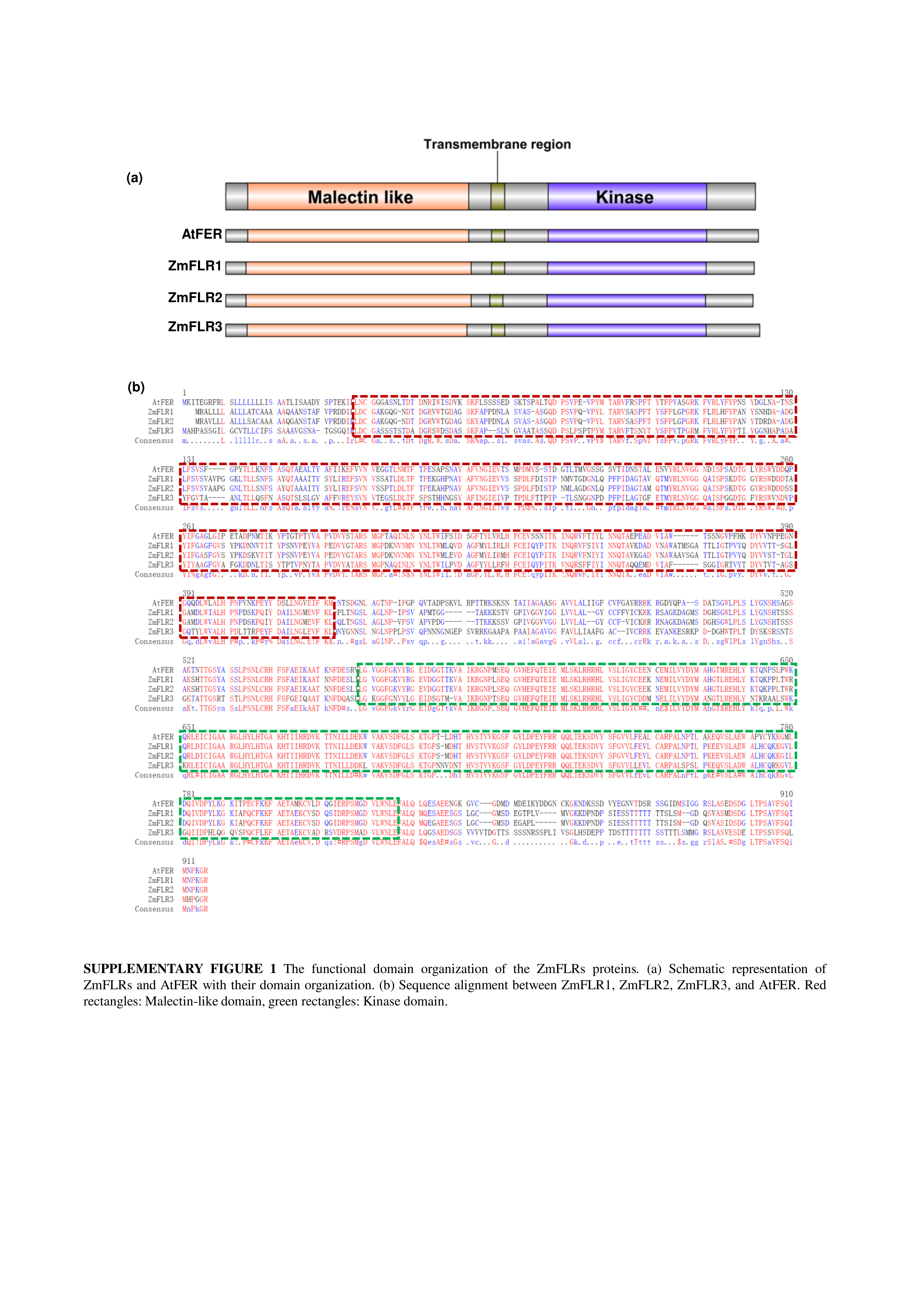

Supplement: Supplementary file 1 — Figure S1 The functional domain organization of the ZmFLR proteins. (a) Schematic representation of ZmFLRs and AtFER with their domain organization. (b) Sequence alignment between ZmFLR1, ZmFLR2, ZmFLR3, and AtFER. Red rectangles, malectin‐like domain; green rectangles, kinase domain [file MPP-23-1331-s005.tif]

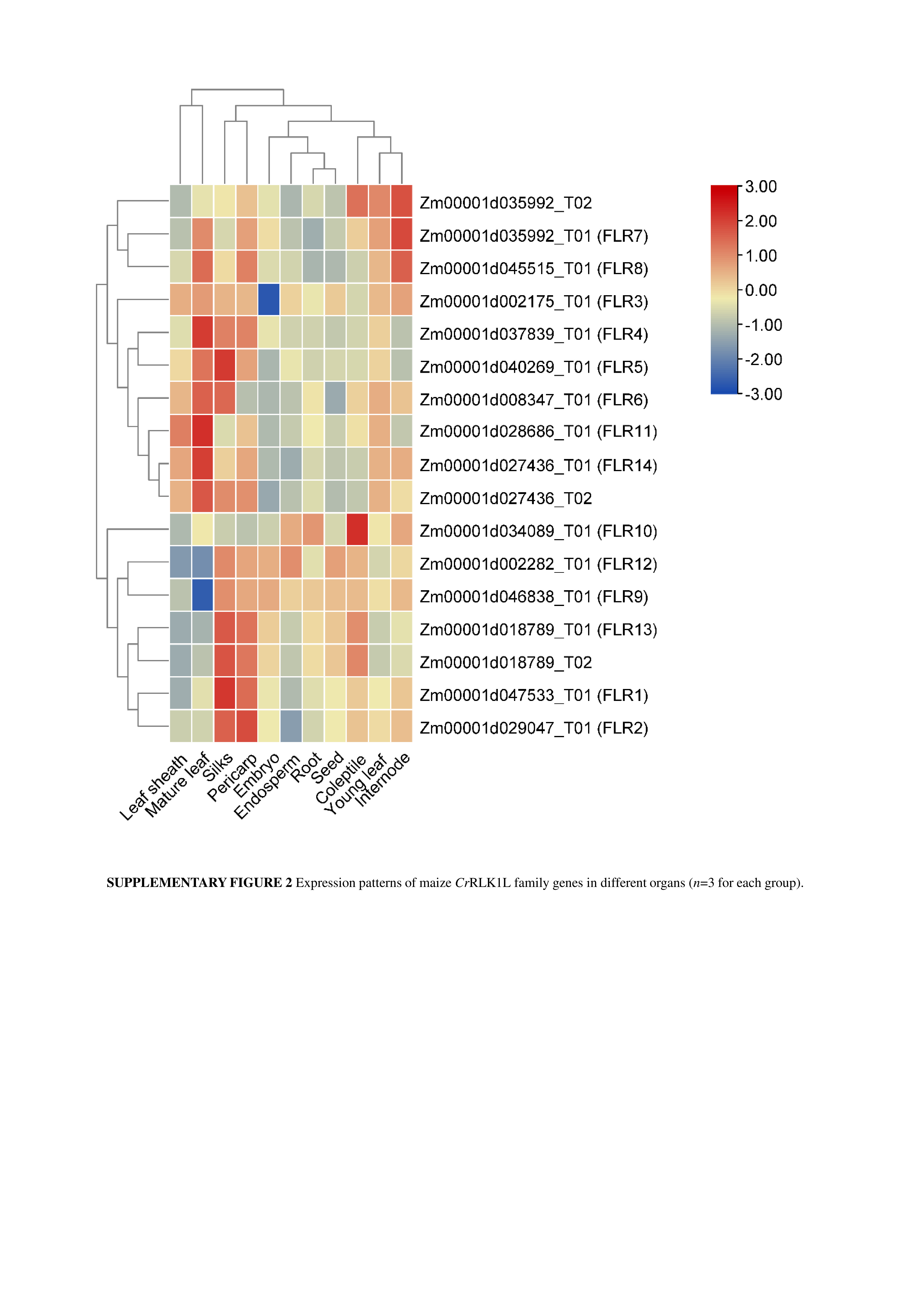

Supplement: Supplementary file 2 — Figure S2 Expression patterns of maize CrRLK1L family genes in different organs (n = 3 for each group) [file MPP-23-1331-s004.tif]

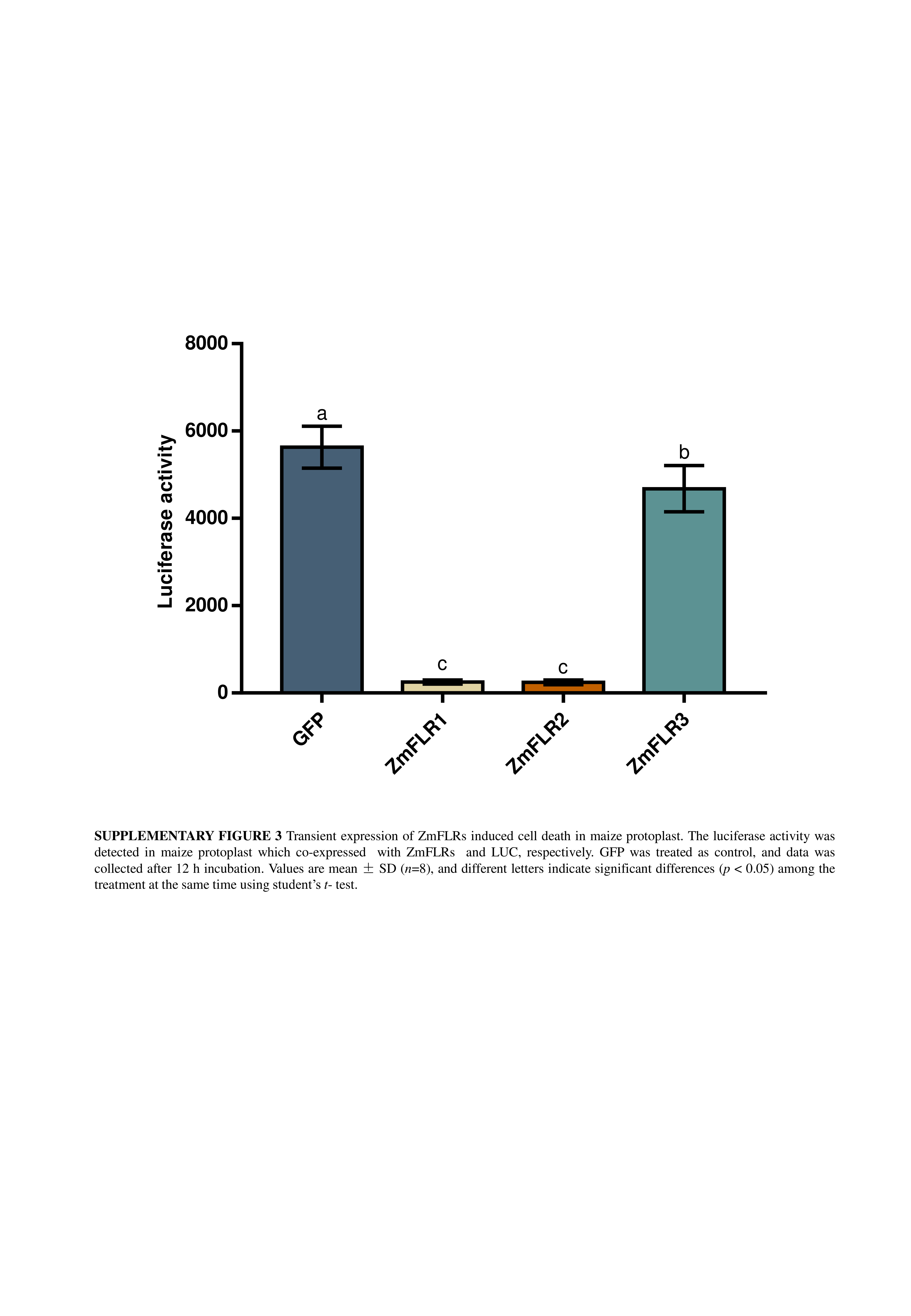

Supplement: Supplementary file 3 — Figure S3 Transient expression of ZmFLRs induced cell death in maize protoplasts. The luciferase activity was detected in maize protoplasts that coexpressed ZmFLRs with LUC. GFP was treated as a control and data were collected after 12 h of incubation. Values are mean ± SD (n = 8), and different letters indicate significant differences (p < 0.05) among the treatments at the same time using Student’s t test [file MPP-23-1331-s001.tif]

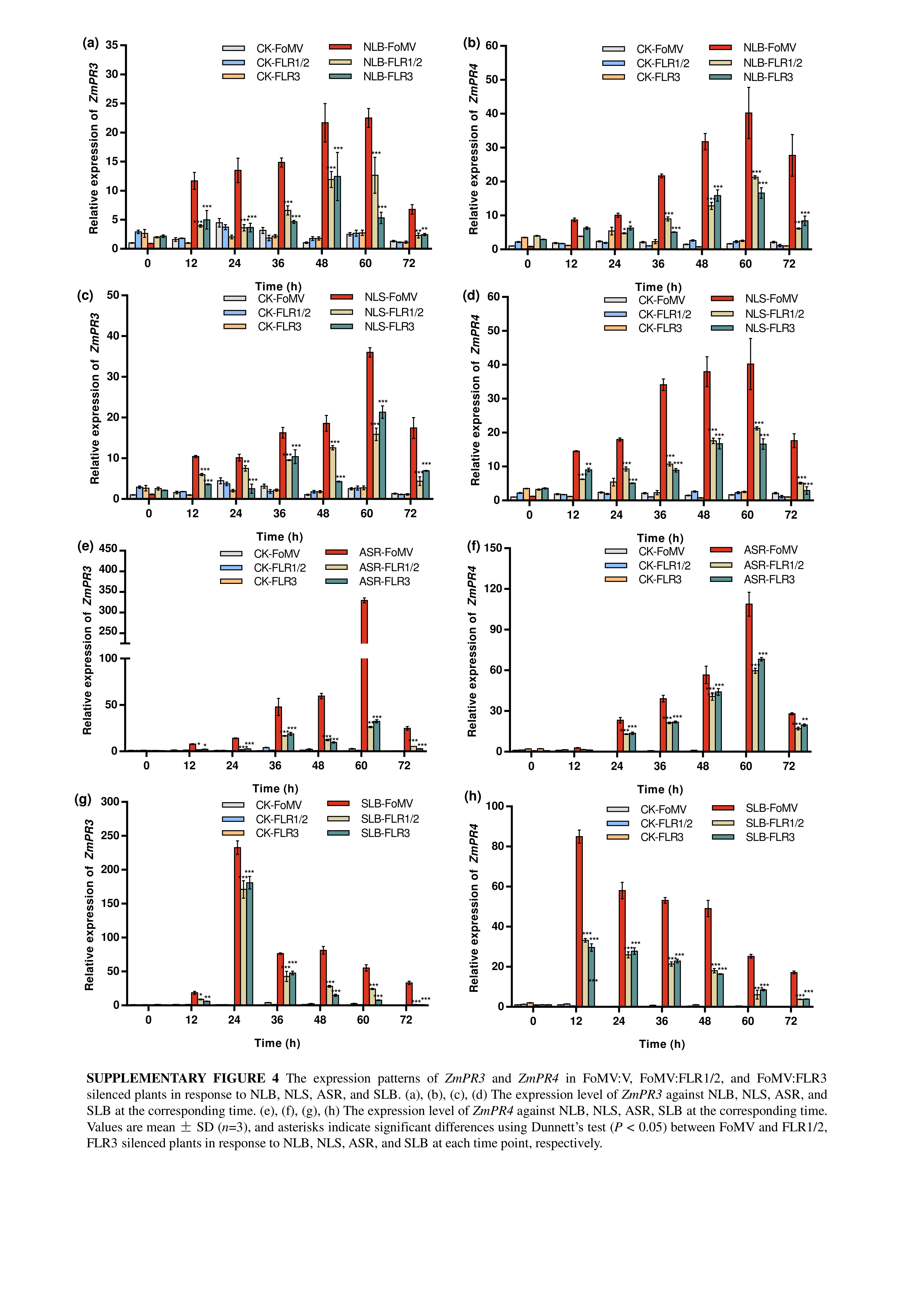

Supplement: Supplementary file 4 — Figure S4 The expression patterns of ZmPR3 and ZmPR4 in FoMV:V, FoMV:FLR1/2, and FoMV:FLR3 silenced plants in response to Setosphaeria turcica (northern corn leaf blight, NLB), Bipolaris zeicola (northern corn leaf spot, NLS), Colletotrichum graminicola (anthracnose stalk rot, ASR), and Bipolaris maydis (southern corn leaf blight, SLB). (a–d) The expression level of ZmPR3 against the four pathogens over time. (e–h) The expression level of ZmPR4 against the four pathogens over time. Values are mean ± SD (n = 3) and asterisks indicate significant differences using Dunnett’s test (p < 0.05) between FoMV and FLR1/2, FLR3 silenced plants in response to the four pathogens at each time point [file MPP-23-1331-s003.tif]
