# Supplementary material for: Maize FERONIA‐like receptor genes are involved in the response of multiple disease resistance in maize
Source: Mol Plant Pathol. 2022 May 21;23(9):1331–45. doi: 10.1111/mpp.13232 (PMC9366073; doi:10.1111/mpp.13232)
Supplement: Supplementary file 5 — Table S1 Primers used in this study [file MPP-23-1331-s002.docx]

**Table S1 Primers used in this study.**

Primers for cloning:

| Gene | Gene ID | Forward primer (5’-3’) | Reverse primer (5’-3’) |
| --- | --- | --- | --- |
| *ZmFLR1* | Zm00001d047533 | ATACACCAAATCGACTCTAGAATGAGGGCCTTGCTGCTGCT | CATGGTACCGGATCCACTAGTCCGCCCCTTGGGGTTCATGA |
| *ZmFLR2* | Zm00001d029047 | ATACACCAAATCGACTCTAGAATGAGGGCCGTGCTGCTGCT | CATGGTACCGGATCCACTAGTCCGCCCCTTGGGGTTCATGA |
| *ZmFLR3* | Zm00001d002175 | ATACACCAAATCGACTCTAGAATGGCACACCCAGCCTCGTC | CATGGTACCGGATCCACTAGTTCTGCCACCTGGATGCATGA |

Primers for VIGS assay:

| Gene | Forward primer (5’-3’) | Reverse primer (5’-3’) |
| --- | --- | --- |
| *ZmFLR1/2* | CCTTAATTAACAACCCTATCCCAAGTGTT | GCTCTAGAGATGGCAAAGATGAAGCATA |
| *ZmFLR3* | CCTTAATTAACCATCTGTTCAGCCTAAC | GCTCTAGA AGTGAGAGGAGTCCAGTG |
| *ZmPDS* | CCTTAATTAACCAAACCGTTCAATGCTGG | GCTCTAGAAGATGGGACGGGAACTTCTC |

Primers for qPCR

| Gene | Gene ID | Forward primer (5’-3’) | Reverse primer (5’-3’) |
| --- | --- | --- | --- |
| *ZmFLR1/2* | Zm00001d047533/Zm00001d029047 | CGACACAGCATACATCTTTGG | TGAATCCAGCATCAACCTG |
| *ZmFLR3* | Zm00001d002175 | CAGATGCTTACTTCGGCGT | GGTGTGTGGATGGAGAGAAAG |
| *ZmPDS* | Zm00001d027936 | TGGAGAAGTTGGTGGGAGTTCC | CAGCATTGAACGGTTTGGGTCA |
| *ZmPR1* | Zm00001d018738 | CCTACGGCGAGAACCTCTT | TCGTAGTACTGCTTCTCGGACA |
| *ZmPR5* | Zm00001d031158 | ATCGGCCGGAATAGGCTCTG | CGCGTACATACAAATGCGTGC |
| *ZmActin* | Zm00001d010159 | GGTTTCGCTGGTGATGATGC | CAATGCCATGCTCAATCGGG |
| *BmActin* | COCHEDRAFT_1197727 | ATCGTCCGTGACATCAAGG | ACGCTCGTTTCCAATGGT |
| *BzActin* | COCCADRAFT_4783 | ATCGTCCGTGACATCAAGG | ACGCTCGTTTCCAATGGT |
| *CgTubulin* | GLRG_01057 | GCAAACCATTCACGGCGAG | GGGCTCAAGGTCAACCAGGA |
